# Supplementary figures and images for: Integrated bulk RNA and single-cell analysis with experimental validation reveal oxidative stress-related diagnostic biomarkers for osteoporosis
Source: PLoS One. 2025 Apr 29;20(4):e0322326. doi: 10.1371/journal.pone.0322326 (PMC12040157; doi:10.1371/journal.pone.0322326)

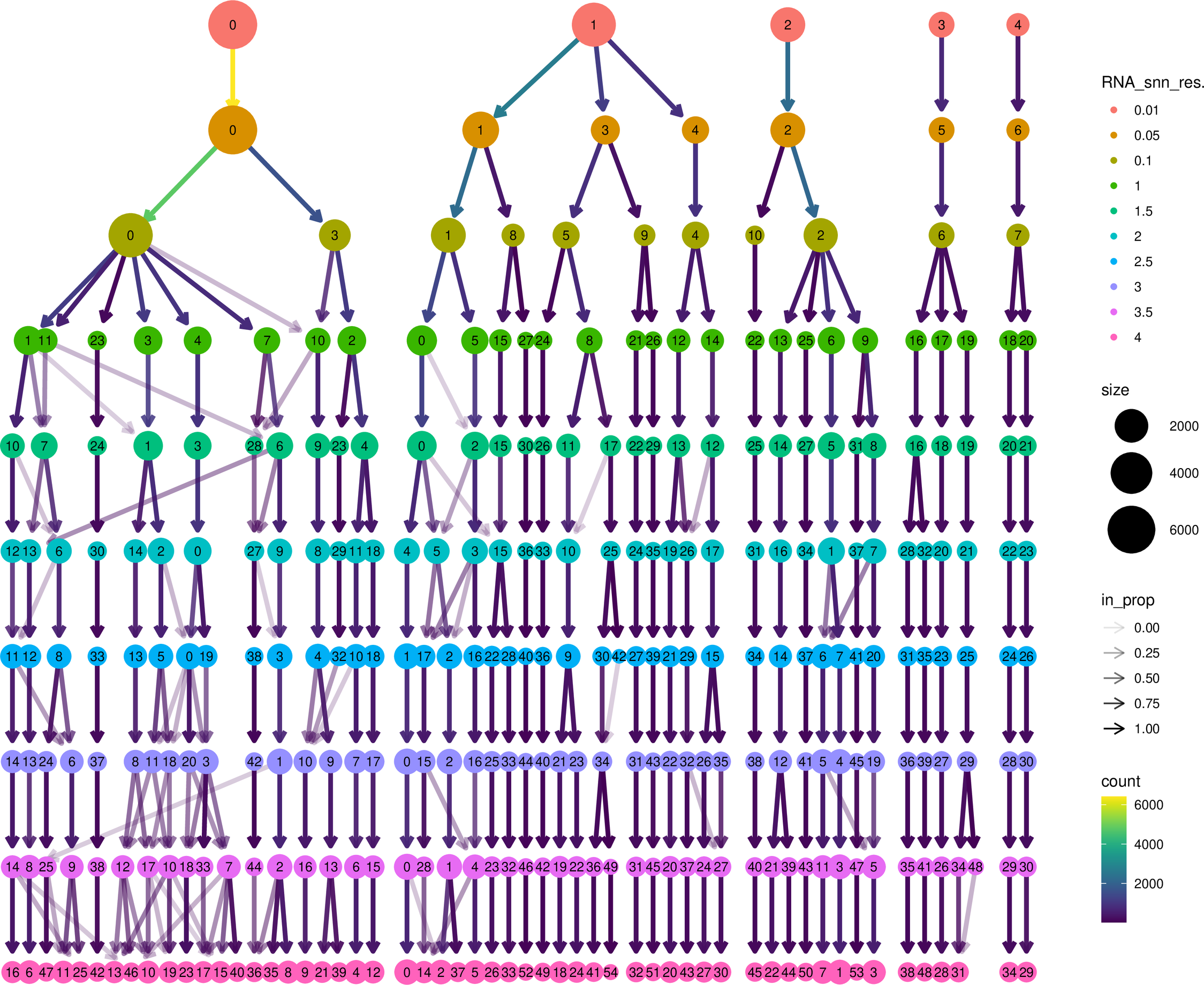

Supplement: S1 Fig — (TIF) [file pone.0322326.s001.tif]

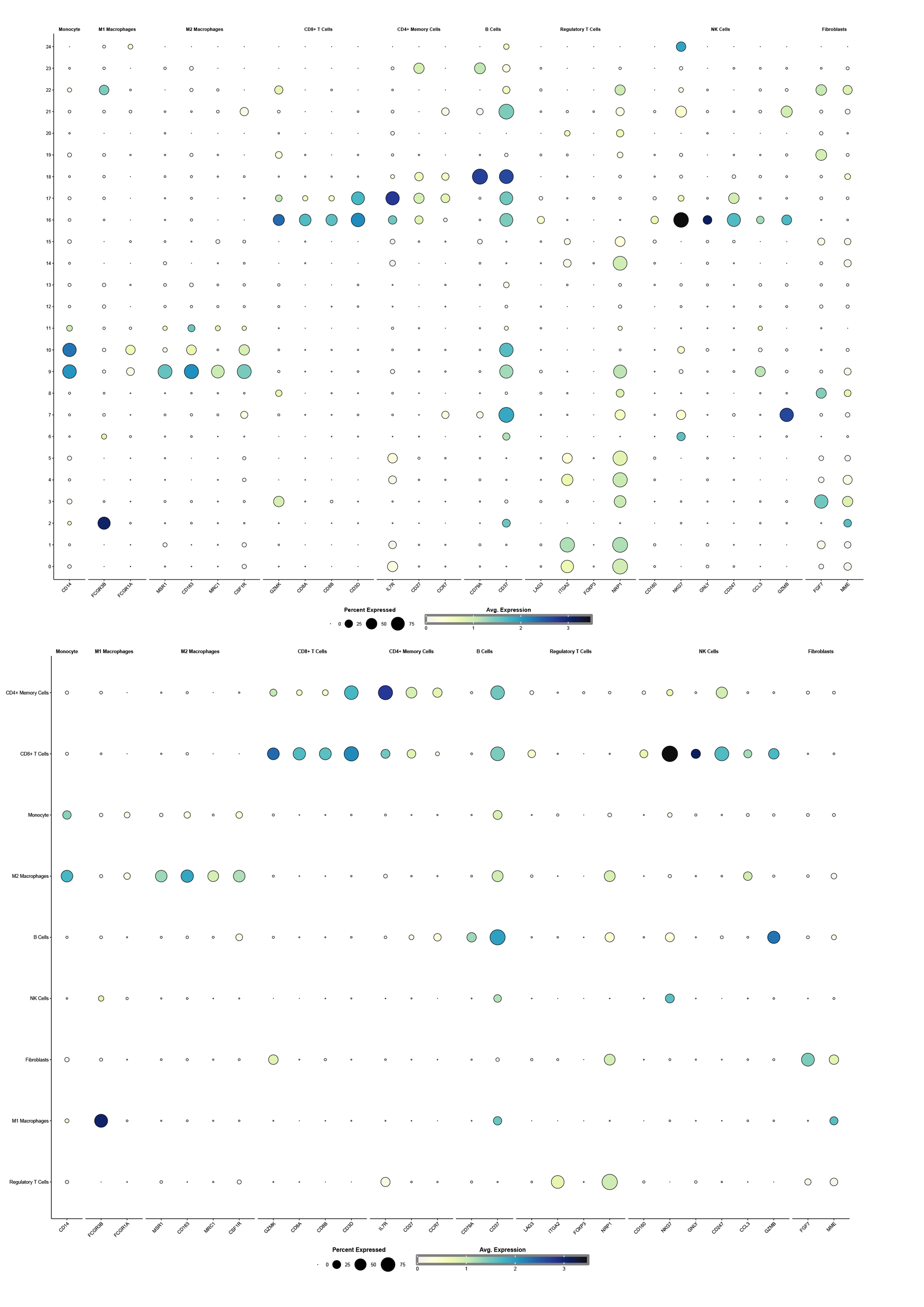

Supplement: S2 Fig — (TIF) [file pone.0322326.s002.tif]

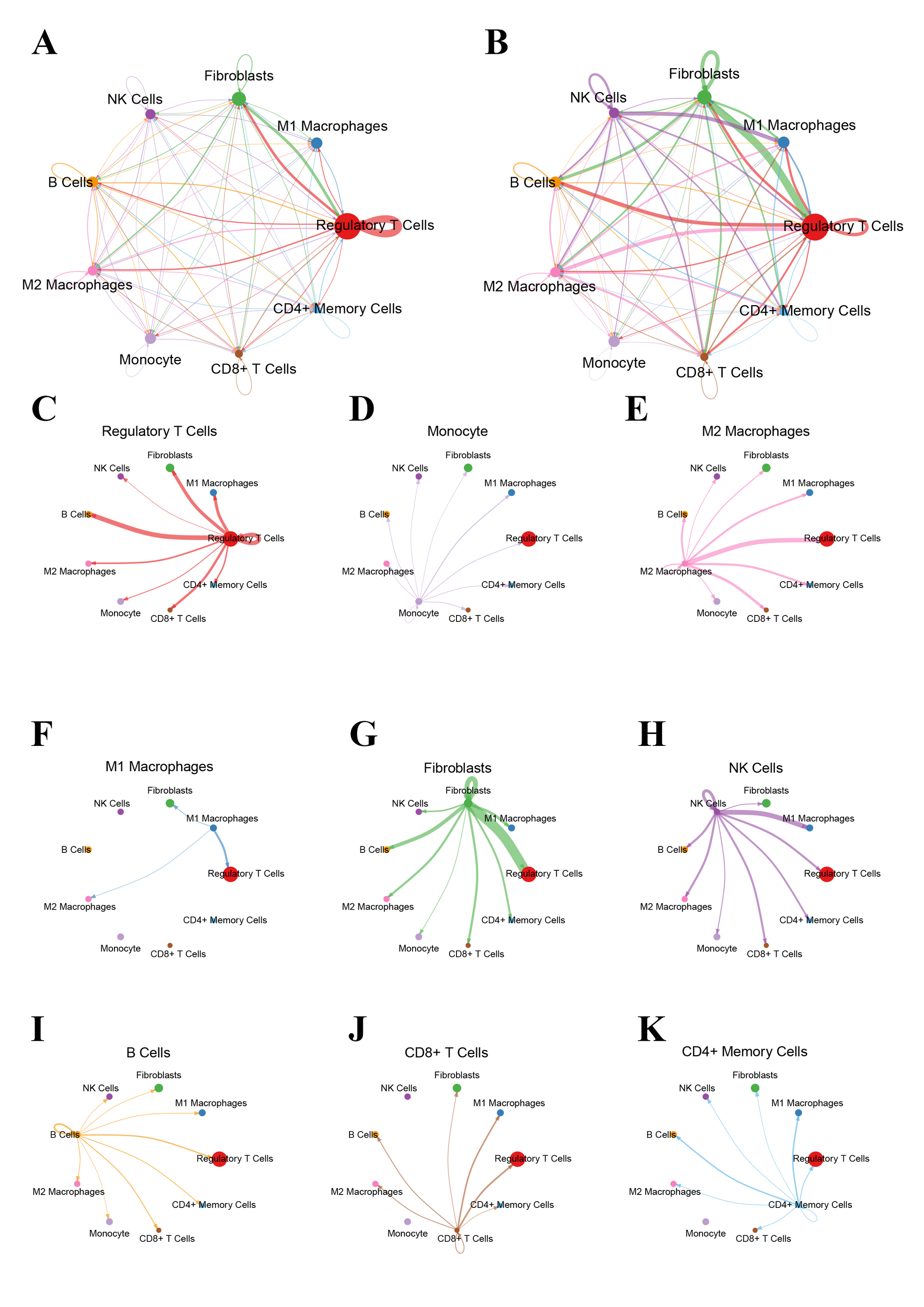

Supplement: S3 Fig — The interaction network of all cell types in normal bone tissues (A) and OP samples (B). The interaction networks among all cell types: (C) regulatory T Cells with other cells; (D) monocytes with other cells; (E) M2 Macrophages with other cells; (F) M1 Macrophages with other cells; (G) Fibroblasts with other cells; (H) NK Cells with other cells; (I) B Cells with other cells; (J) CD8+ T Cells with other cells; (K) CD4+ Memory T Cells with other cells. (TIF) [file pone.0322326.s003.tif]

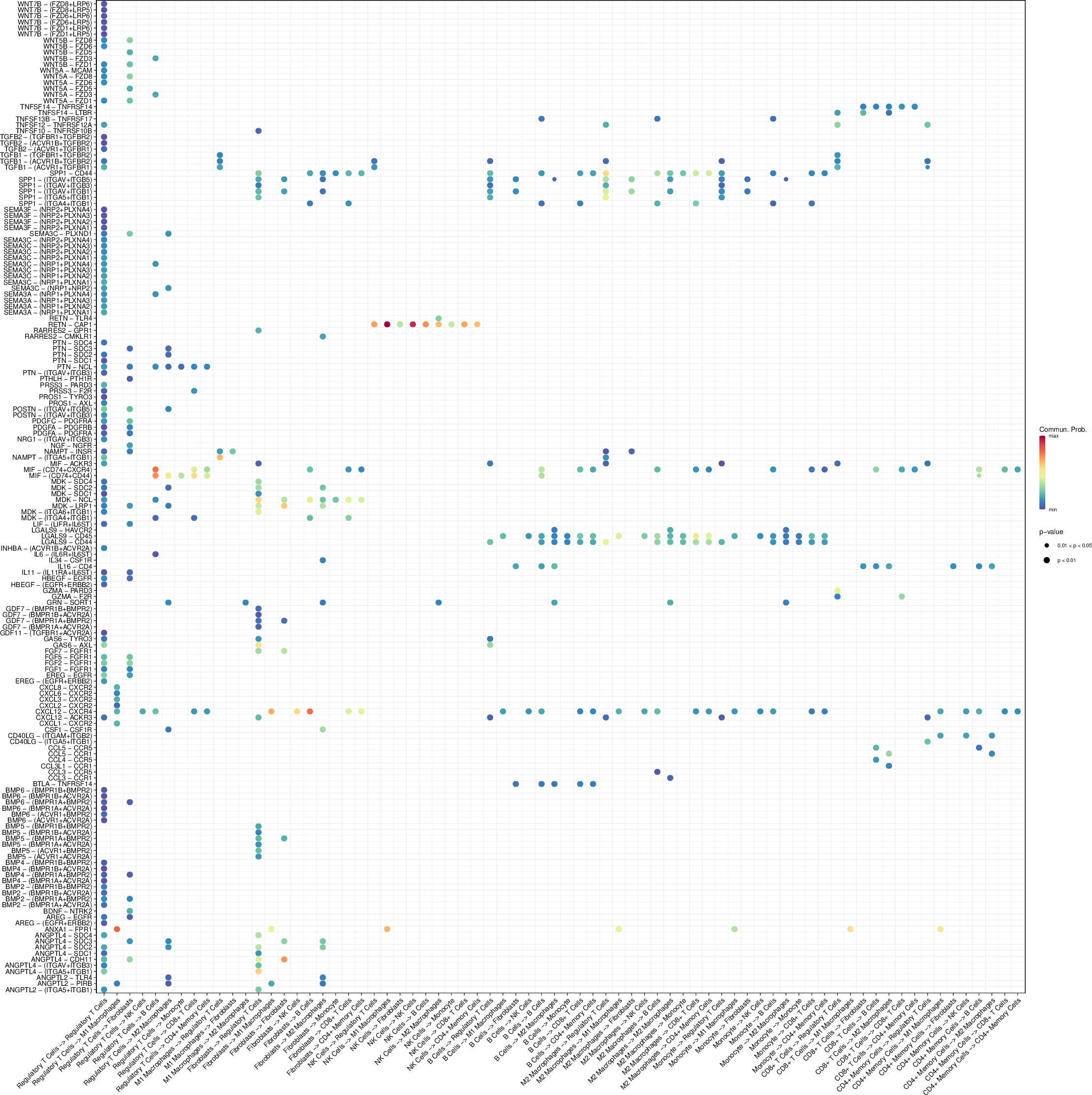

Supplement: S4 Fig — (TIF) [file pone.0322326.s004.tif]

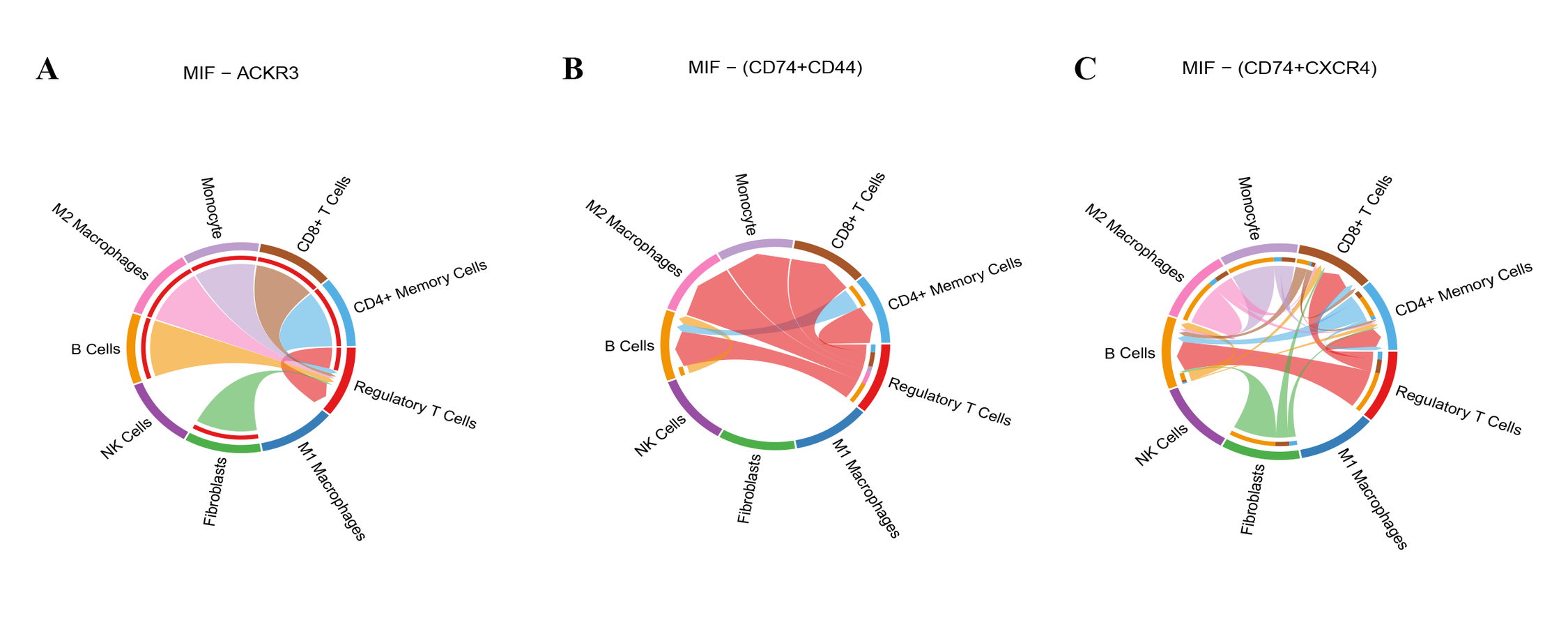

Supplement: S5 Fig — (A) The receptor of ACKR3. (B) The receptor and ligand interaction of CD74 and CD44. (C) The receptor and ligand interaction of CD74 and CXCR4. (TIF) [file pone.0322326.s005.tif]

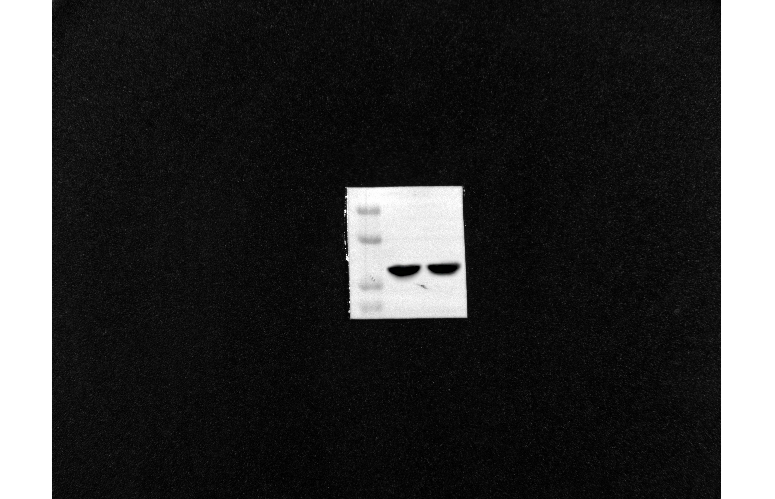

Supplement: S1 File — (ZIP) [file pone.0322326.s006.zip › ú¿1ú⌐+ú¿3ú⌐WB/ú¿1ú⌐/╩╡╤Θ╞▀ú¿1ú⌐ GAPDH merge(1).tif]

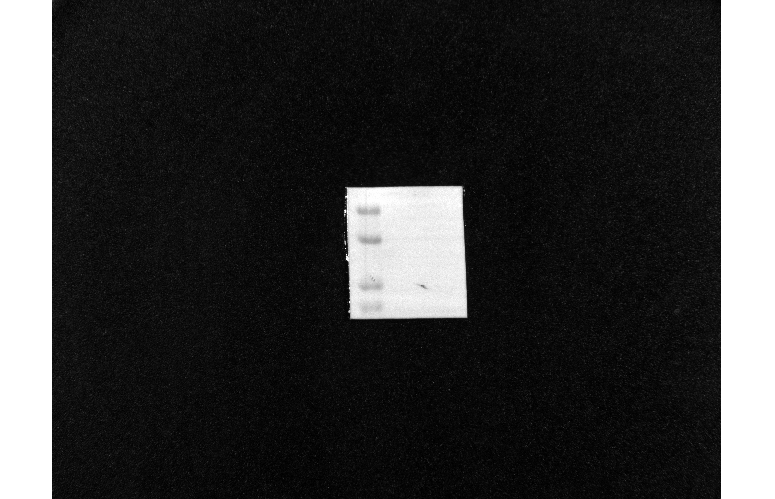

Supplement: S1 File — (ZIP) [file pone.0322326.s006.zip › ú¿1ú⌐+ú¿3ú⌐WB/ú¿1ú⌐/╩╡╤Θ╞▀ú¿1ú⌐ GAPDH ░╫╣Γ(1).tif]

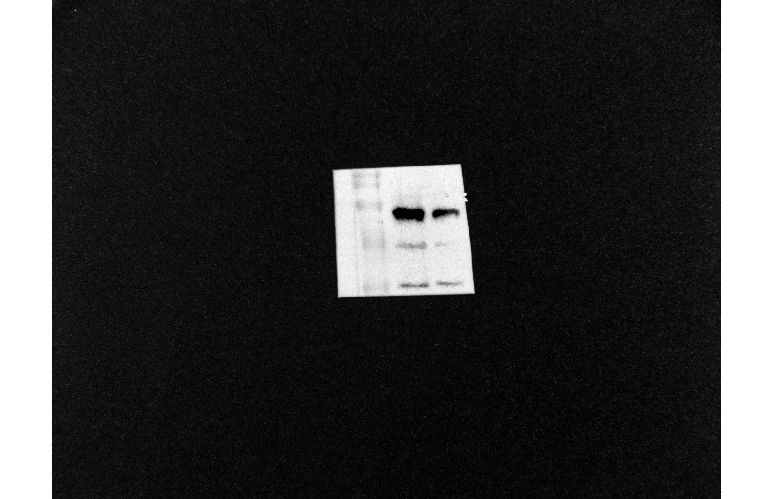

Supplement: S1 File — (ZIP) [file pone.0322326.s006.zip › ú¿1ú⌐+ú¿3ú⌐WB/ú¿1ú⌐/╩╡╤Θ╞▀ú¿1ú⌐COL4A2 merge(1).tif]

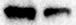

Supplement: S1 File — (ZIP) [file pone.0322326.s006.zip › ú¿1ú⌐+ú¿3ú⌐WB/ú¿1ú⌐/╩╡╤Θ╞▀ú¿1ú⌐COL4A2 ╜╪(1).tif]

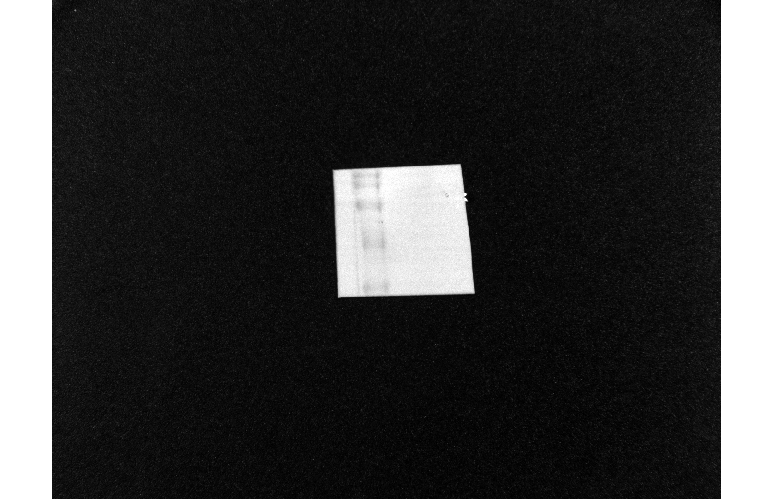

Supplement: S1 File — (ZIP) [file pone.0322326.s006.zip › ú¿1ú⌐+ú¿3ú⌐WB/ú¿1ú⌐/╩╡╤Θ╞▀ú¿1ú⌐COL4A2 ░╫╣Γ(1).tif]

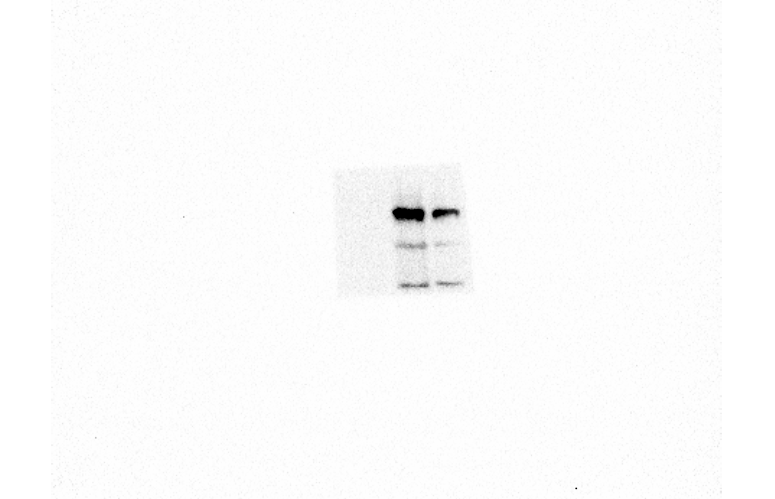

Supplement: S1 File — (ZIP) [file pone.0322326.s006.zip › ú¿1ú⌐+ú¿3ú⌐WB/ú¿1ú⌐/╩╡╤Θ╞▀ú¿1ú⌐COL4A2 ╫╧═Γ(1).tif]

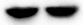

Supplement: S1 File — (ZIP) [file pone.0322326.s006.zip › ú¿1ú⌐+ú¿3ú⌐WB/ú¿1ú⌐/╩╡╤Θ╞▀ú¿1ú⌐GAPDH ╜╪(1).tif]

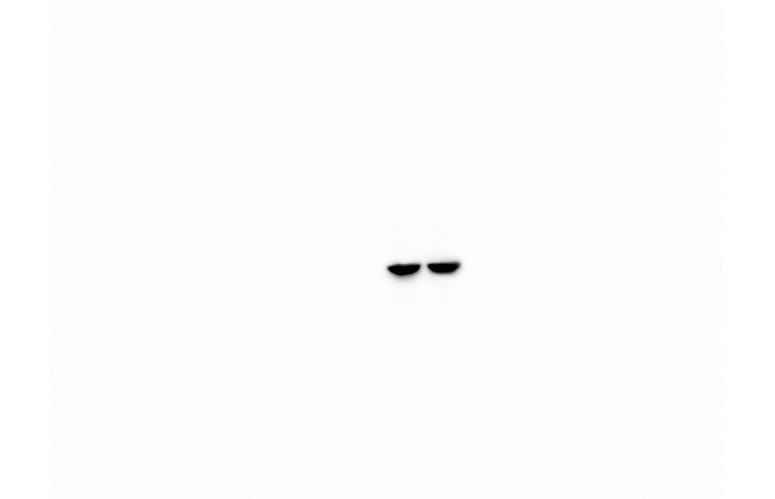

Supplement: S1 File — (ZIP) [file pone.0322326.s006.zip › ú¿1ú⌐+ú¿3ú⌐WB/ú¿1ú⌐/╩╡╤Θ╞▀ú¿1ú⌐GAPDH ╫╧═Γ(1).tif]

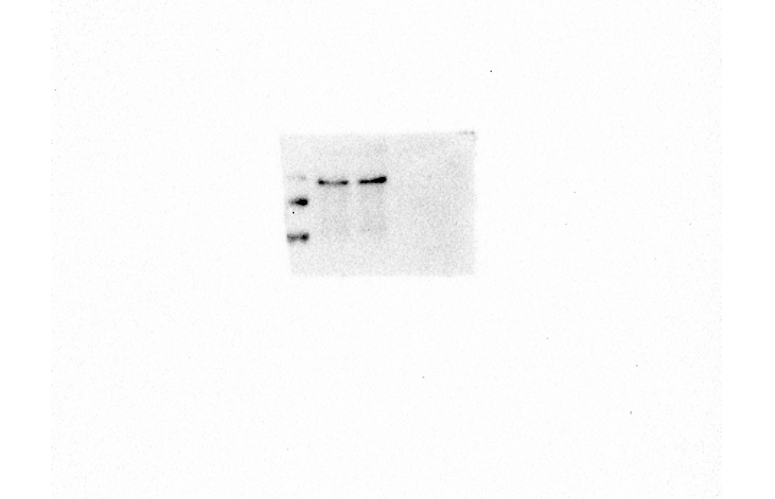

Supplement: S1 File — (ZIP) [file pone.0322326.s006.zip › ú¿1ú⌐+ú¿3ú⌐WB/ú¿3ú⌐/╩╡╤Θ╞▀ú¿3ú⌐CHRM2 ╘¡.tif]

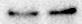

Supplement: S1 File — (ZIP) [file pone.0322326.s006.zip › ú¿1ú⌐+ú¿3ú⌐WB/ú¿3ú⌐/╩╡╤Θ╞▀ú¿3ú⌐CHRM2 ╜╪.tif]

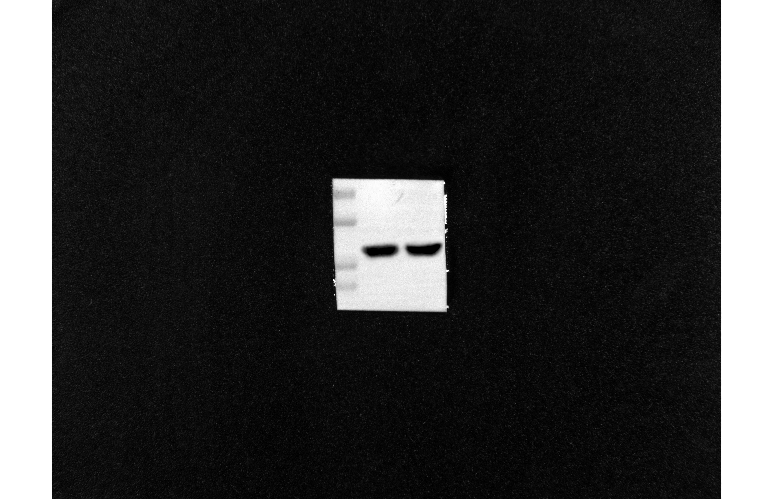

Supplement: S1 File — (ZIP) [file pone.0322326.s006.zip › ú¿1ú⌐+ú¿3ú⌐WB/ú¿3ú⌐/╩╡╤Θ╞▀ú¿3ú⌐GAPDH merge.tif]

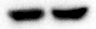

Supplement: S1 File — (ZIP) [file pone.0322326.s006.zip › ú¿1ú⌐+ú¿3ú⌐WB/ú¿3ú⌐/╩╡╤Θ╞▀ú¿3ú⌐GAPDH ╜╪.tif]

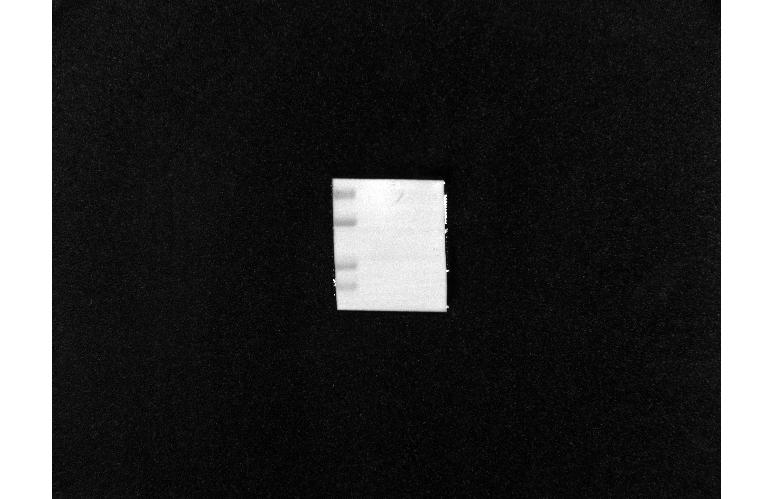

Supplement: S1 File — (ZIP) [file pone.0322326.s006.zip › ú¿1ú⌐+ú¿3ú⌐WB/ú¿3ú⌐/╩╡╤Θ╞▀ú¿3ú⌐GAPDH ░╫╣Γ.tif]

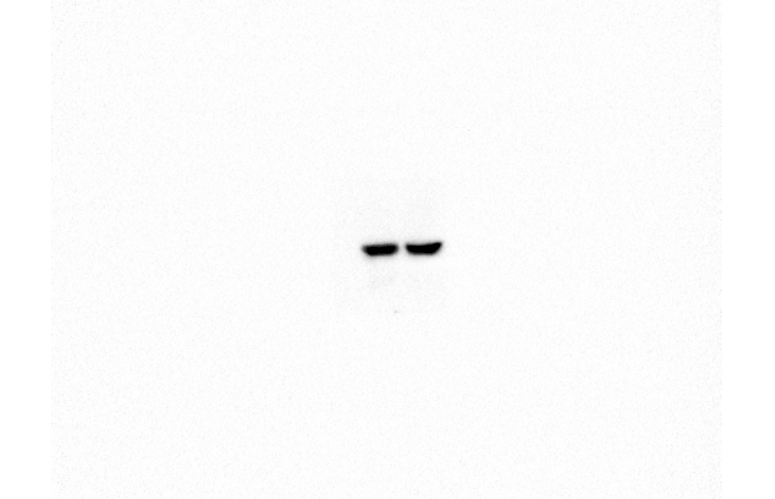

Supplement: S1 File — (ZIP) [file pone.0322326.s006.zip › ú¿1ú⌐+ú¿3ú⌐WB/ú¿3ú⌐/╩╡╤Θ╞▀ú¿3ú⌐GAPDH ╫╧═Γ.tif]

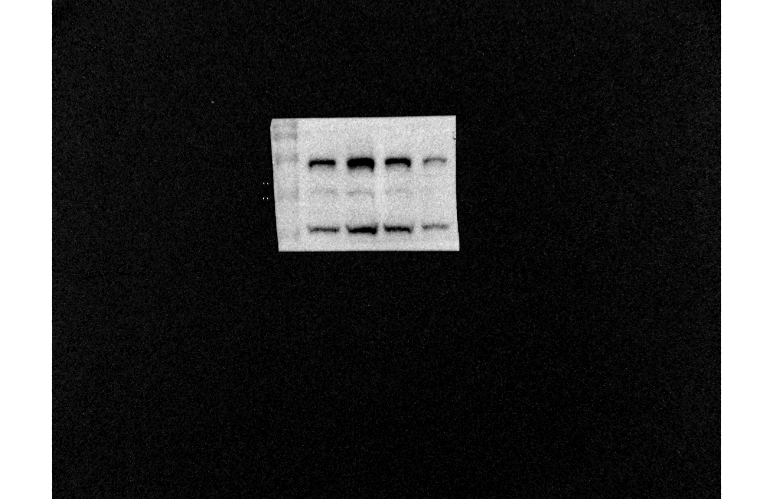

Supplement: S1 File — (ZIP) [file pone.0322326.s006.zip › ú¿2ú⌐WB/╩╡╤Θ╬σú¿2ú⌐COL4A2 merge.tif]

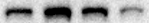

Supplement: S1 File — (ZIP) [file pone.0322326.s006.zip › ú¿2ú⌐WB/╩╡╤Θ╬σú¿2ú⌐COL4A2 ╜╪.tif]

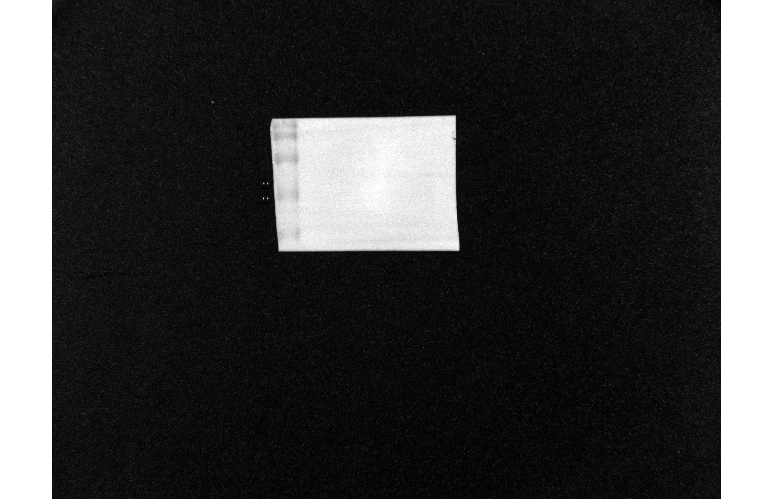

Supplement: S1 File — (ZIP) [file pone.0322326.s006.zip › ú¿2ú⌐WB/╩╡╤Θ╬σú¿2ú⌐COL4A2 ░╫╣Γ.tif]

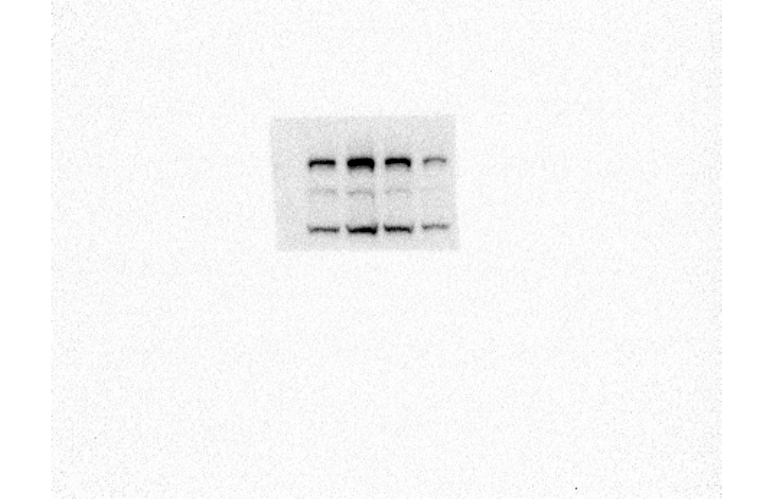

Supplement: S1 File — (ZIP) [file pone.0322326.s006.zip › ú¿2ú⌐WB/╩╡╤Θ╬σú¿2ú⌐COL4A2 ╫╧═Γ.tif]

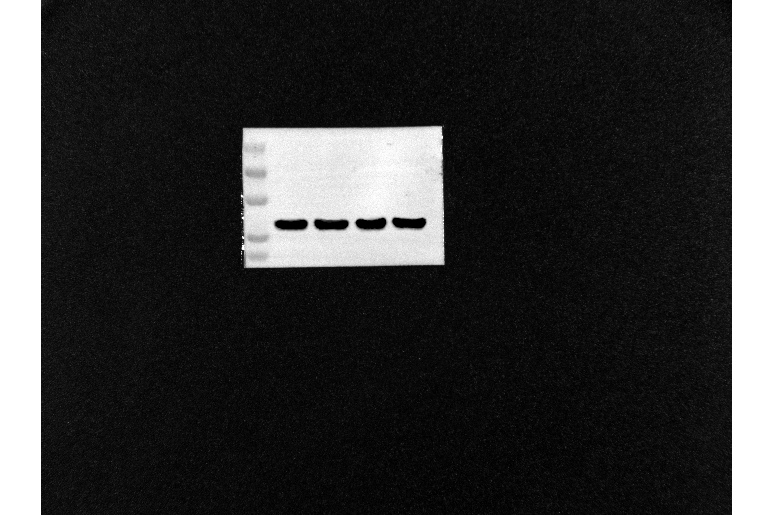

Supplement: S1 File — (ZIP) [file pone.0322326.s006.zip › ú¿2ú⌐WB/╩╡╤Θ╬σú¿2ú⌐GAPDH merge.tif]

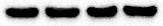

Supplement: S1 File — (ZIP) [file pone.0322326.s006.zip › ú¿2ú⌐WB/╩╡╤Θ╬σú¿2ú⌐GAPDH ╜╪.tif]

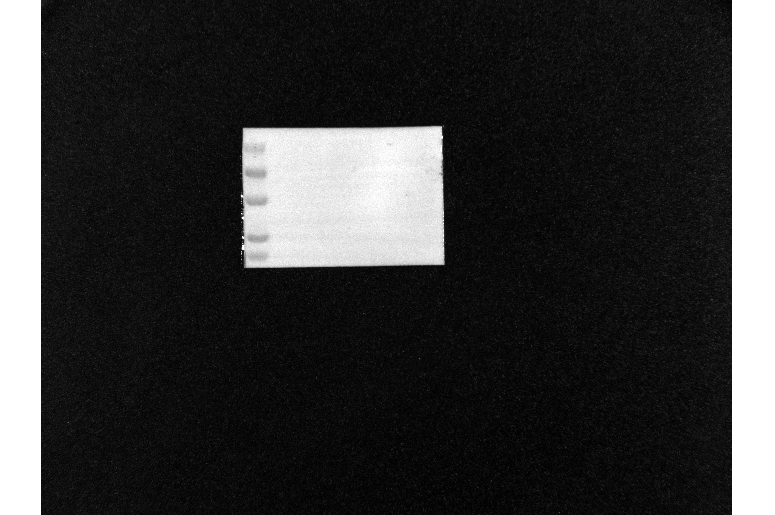

Supplement: S1 File — (ZIP) [file pone.0322326.s006.zip › ú¿2ú⌐WB/╩╡╤Θ╬σú¿2ú⌐GAPDH ░╫╣Γ.tif]

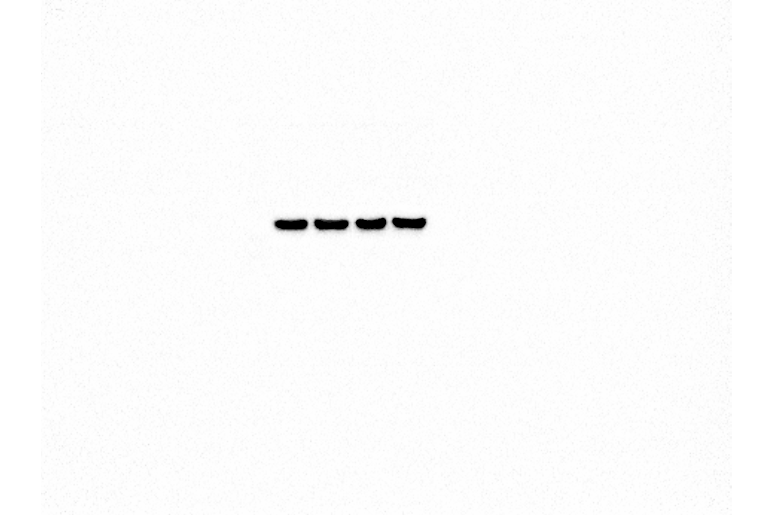

Supplement: S1 File — (ZIP) [file pone.0322326.s006.zip › ú¿2ú⌐WB/╩╡╤Θ╬σú¿2ú⌐GAPDH ╫╧═Γ.tif]

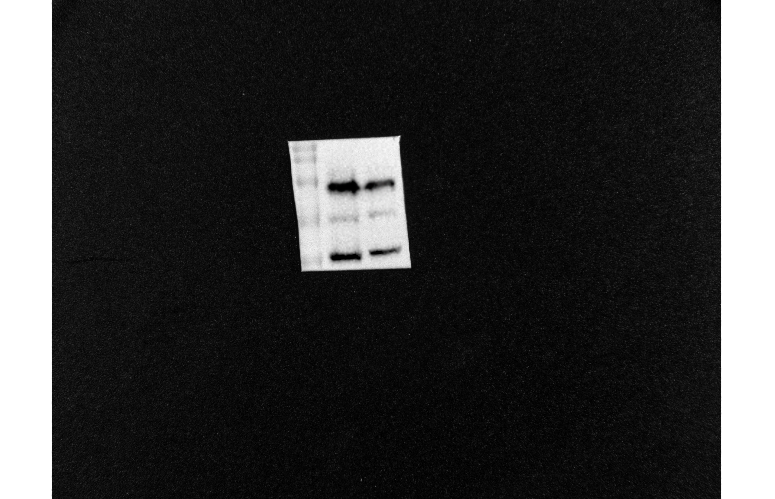

Supplement: S1 File — (ZIP) [file pone.0322326.s006.zip › ú¿2ú⌐WB/╩╡╤Θ┴∙ú¿2ú⌐COL4A2 merge.tif]

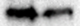

Supplement: S1 File — (ZIP) [file pone.0322326.s006.zip › ú¿2ú⌐WB/╩╡╤Θ┴∙ú¿2ú⌐COL4A2 ╜╪.tif]

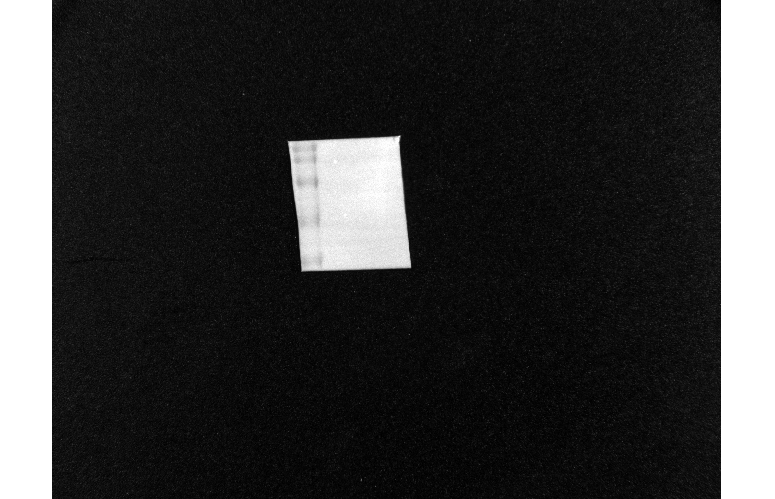

Supplement: S1 File — (ZIP) [file pone.0322326.s006.zip › ú¿2ú⌐WB/╩╡╤Θ┴∙ú¿2ú⌐COL4A2 ░╫╣Γ.tif]

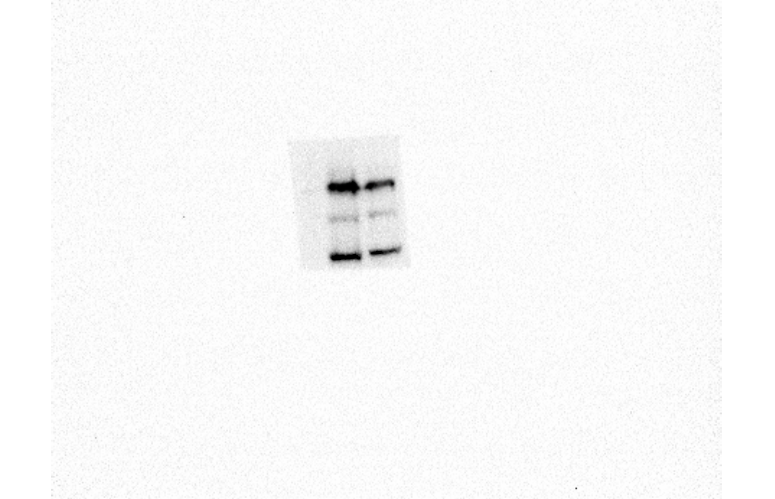

Supplement: S1 File — (ZIP) [file pone.0322326.s006.zip › ú¿2ú⌐WB/╩╡╤Θ┴∙ú¿2ú⌐COL4A2 ╫╧═Γ.tif]

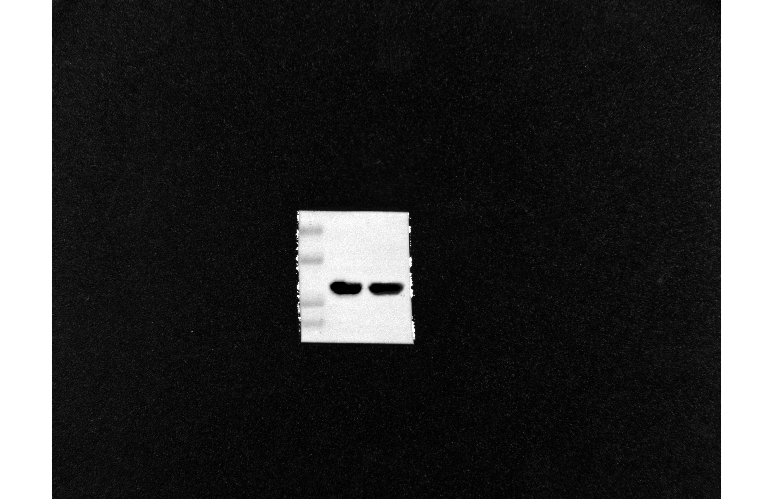

Supplement: S1 File — (ZIP) [file pone.0322326.s006.zip › ú¿2ú⌐WB/╩╡╤Θ┴∙ú¿2ú⌐GAPDH merge.tif]

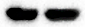

Supplement: S1 File — (ZIP) [file pone.0322326.s006.zip › ú¿2ú⌐WB/╩╡╤Θ┴∙ú¿2ú⌐GAPDH ╜╪.tif]

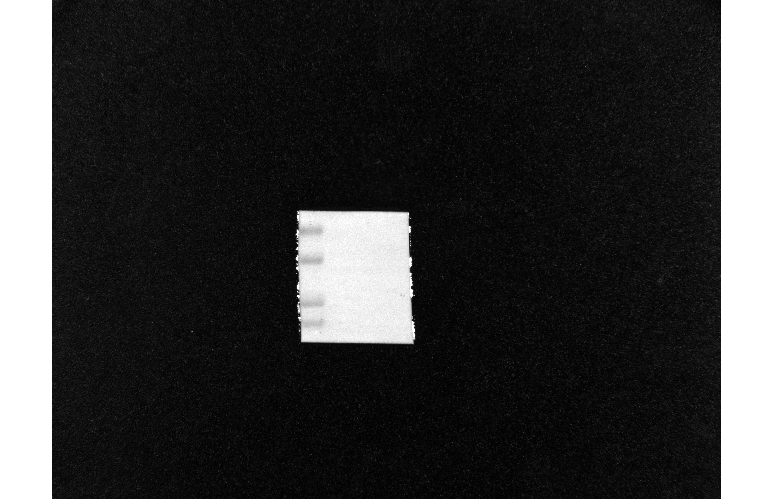

Supplement: S1 File — (ZIP) [file pone.0322326.s006.zip › ú¿2ú⌐WB/╩╡╤Θ┴∙ú¿2ú⌐GAPDH ░╫╣Γ.tif]

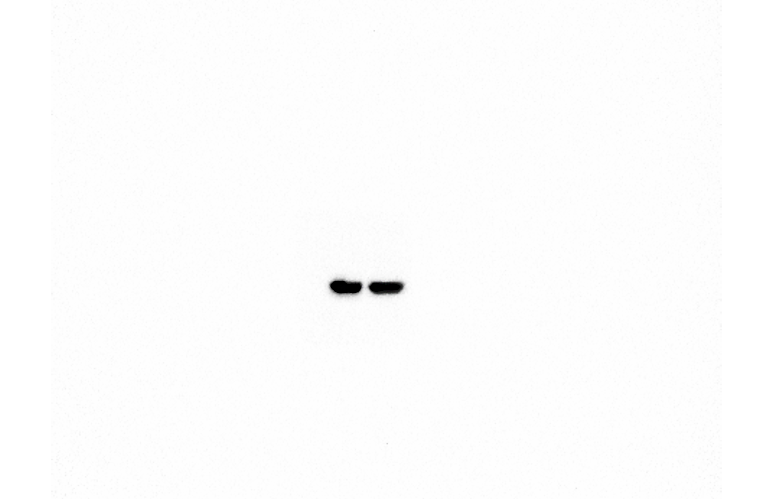

Supplement: S1 File — (ZIP) [file pone.0322326.s006.zip › ú¿2ú⌐WB/╩╡╤Θ┴∙ú¿2ú⌐GAPDH ╫╧═Γ.tif]

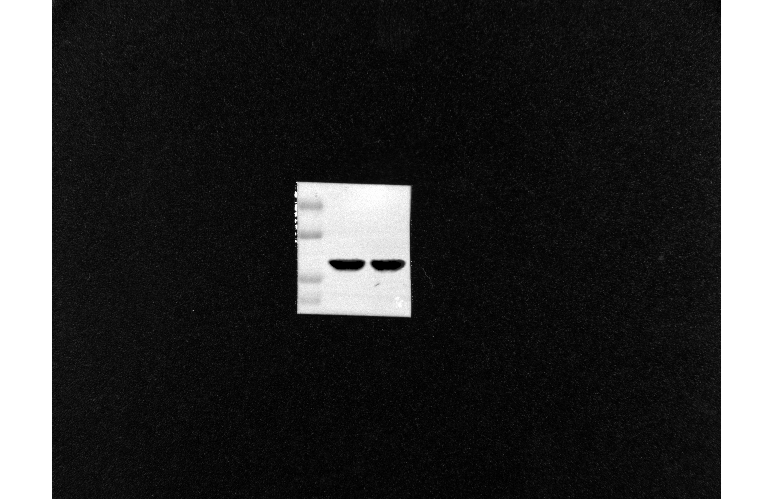

Supplement: S1 File — (ZIP) [file pone.0322326.s006.zip › ú¿6ú⌐WB/╩╡╤Θ╦─ú¿6ú⌐GAPDH merge.tif]

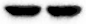

Supplement: S1 File — (ZIP) [file pone.0322326.s006.zip › ú¿6ú⌐WB/╩╡╤Θ╦─ú¿6ú⌐GAPDH ╜╪.tif]

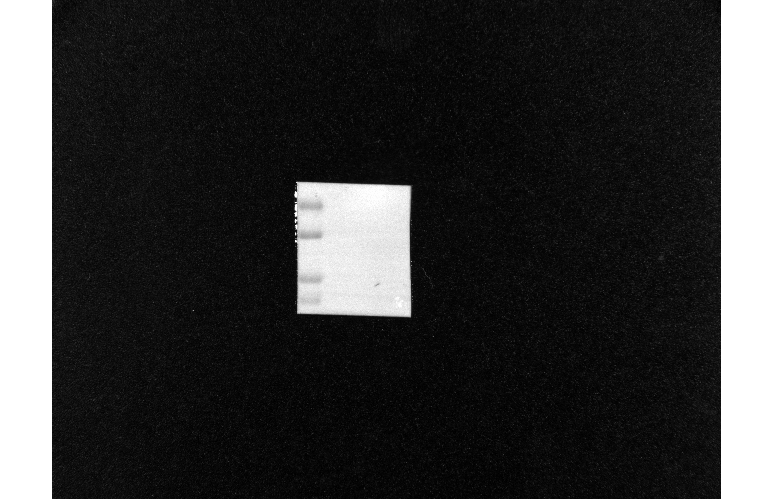

Supplement: S1 File — (ZIP) [file pone.0322326.s006.zip › ú¿6ú⌐WB/╩╡╤Θ╦─ú¿6ú⌐GAPDH ░╫╣Γ.tif]

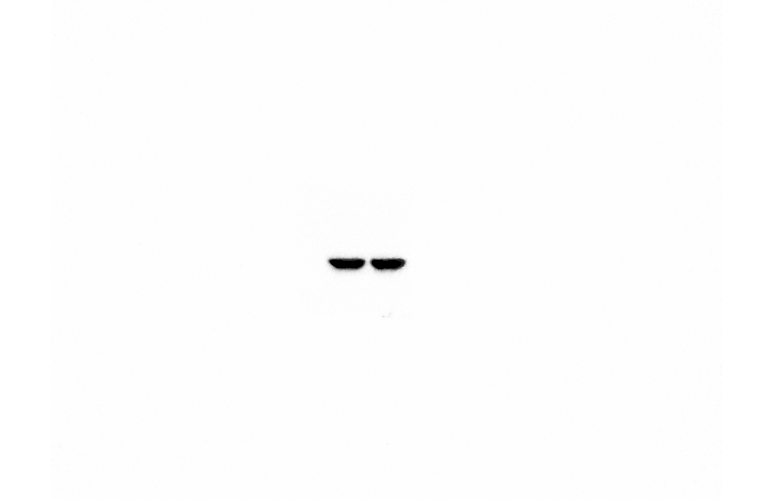

Supplement: S1 File — (ZIP) [file pone.0322326.s006.zip › ú¿6ú⌐WB/╩╡╤Θ╦─ú¿6ú⌐GAPDH ╫╧═Γ.tif]

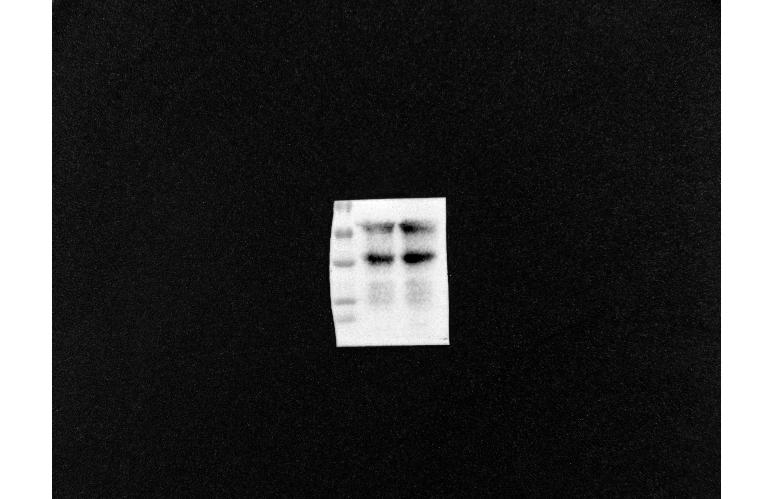

Supplement: S1 File — (ZIP) [file pone.0322326.s006.zip › ú¿6ú⌐WB/╩╡╤Θ╦─ú¿6ú⌐OPN merge.tif]

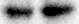

Supplement: S1 File — (ZIP) [file pone.0322326.s006.zip › ú¿6ú⌐WB/╩╡╤Θ╦─ú¿6ú⌐OPN ╜╪.tif]

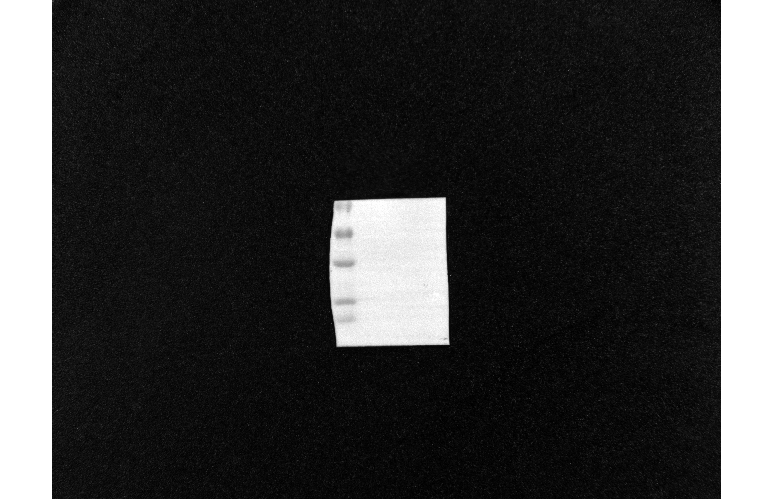

Supplement: S1 File — (ZIP) [file pone.0322326.s006.zip › ú¿6ú⌐WB/╩╡╤Θ╦─ú¿6ú⌐OPN ░╫╣Γ.tif]

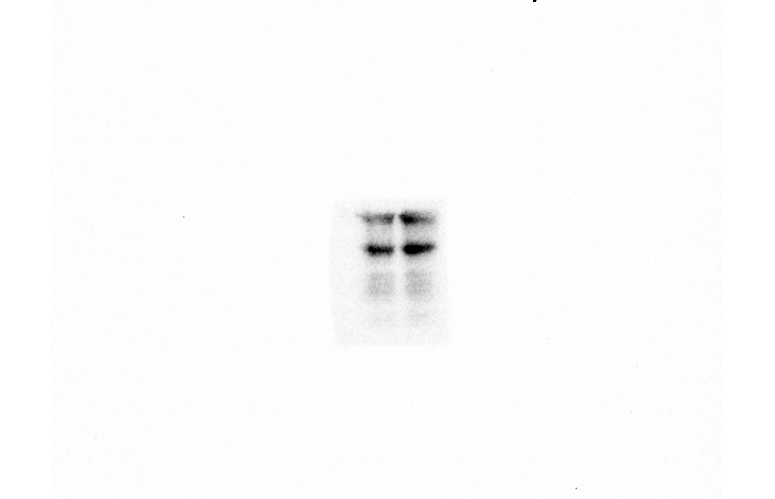

Supplement: S1 File — (ZIP) [file pone.0322326.s006.zip › ú¿6ú⌐WB/╩╡╤Θ╦─ú¿6ú⌐OPN ╫╧═Γ.tif]

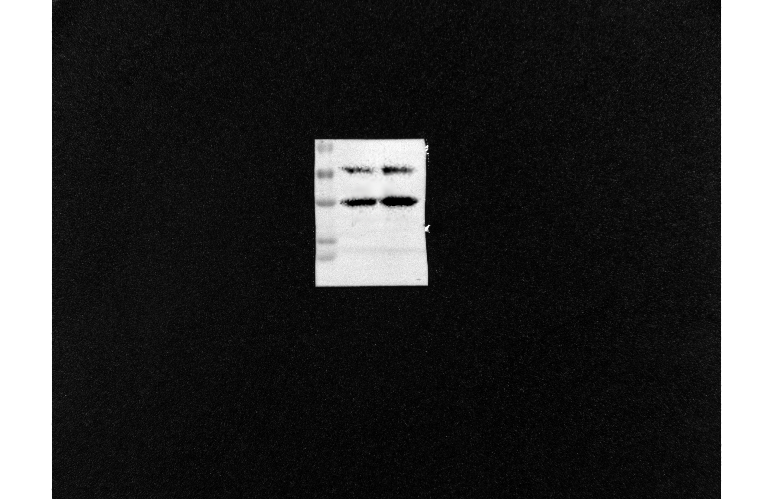

Supplement: S1 File — (ZIP) [file pone.0322326.s006.zip › ú¿6ú⌐WB/╩╡╤Θ╦─ú¿6ú⌐Osterix merge.tif]

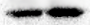

Supplement: S1 File — (ZIP) [file pone.0322326.s006.zip › ú¿6ú⌐WB/╩╡╤Θ╦─ú¿6ú⌐Osterix ╜╪.tif]

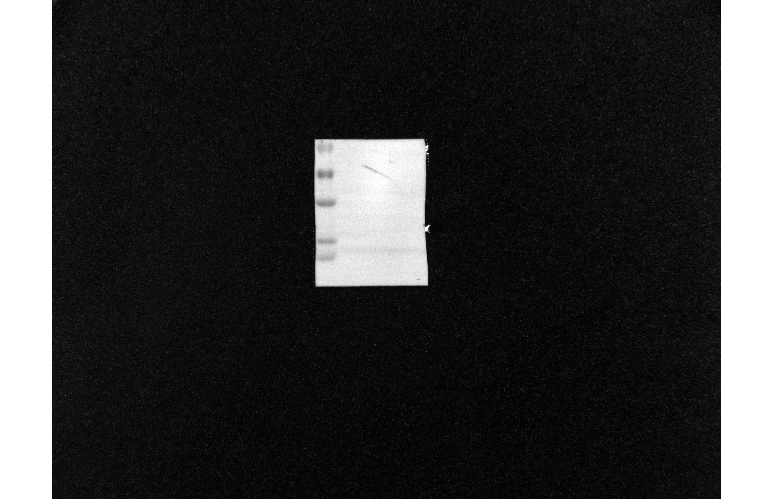

Supplement: S1 File — (ZIP) [file pone.0322326.s006.zip › ú¿6ú⌐WB/╩╡╤Θ╦─ú¿6ú⌐Osterix ░╫╣Γ.tif]

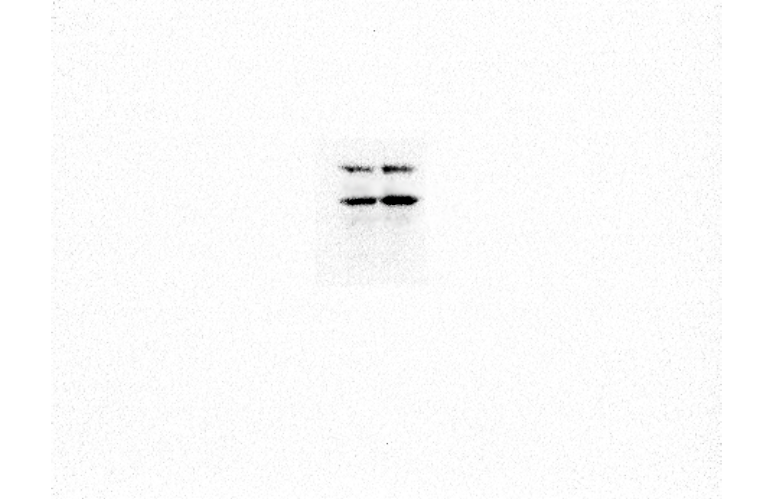

Supplement: S1 File — (ZIP) [file pone.0322326.s006.zip › ú¿6ú⌐WB/╩╡╤Θ╦─ú¿6ú⌐Osterix ╫╧═Γ.tif]

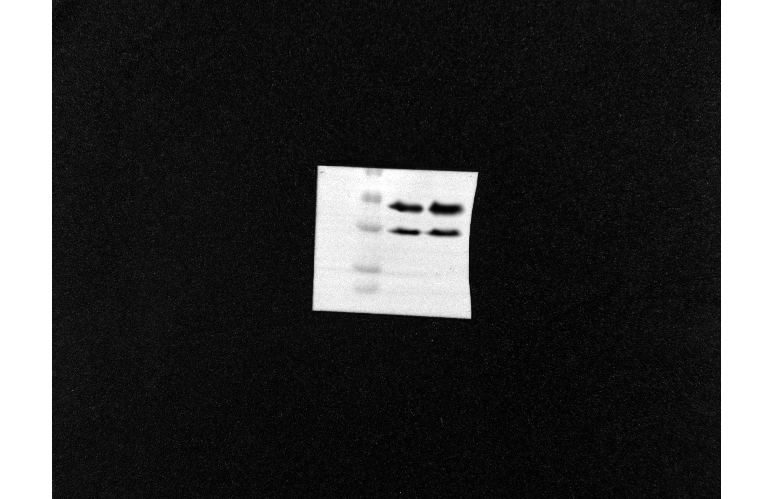

Supplement: S1 File — (ZIP) [file pone.0322326.s006.zip › ú¿6ú⌐WB/╩╡╤Θ╦─ú¿6ú⌐RUNX2 merge.tif]

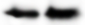

Supplement: S1 File — (ZIP) [file pone.0322326.s006.zip › ú¿6ú⌐WB/╩╡╤Θ╦─ú¿6ú⌐RUNX2 ╜╪.tif]

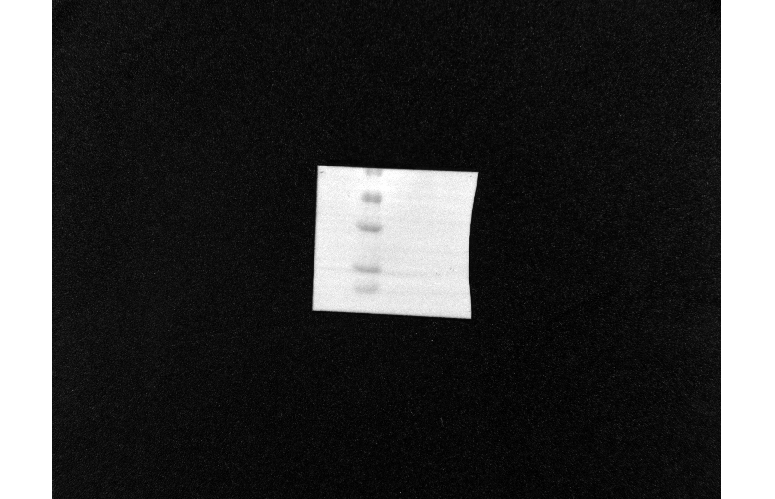

Supplement: S1 File — (ZIP) [file pone.0322326.s006.zip › ú¿6ú⌐WB/╩╡╤Θ╦─ú¿6ú⌐RUNX2 ░╫╣Γ.tif]

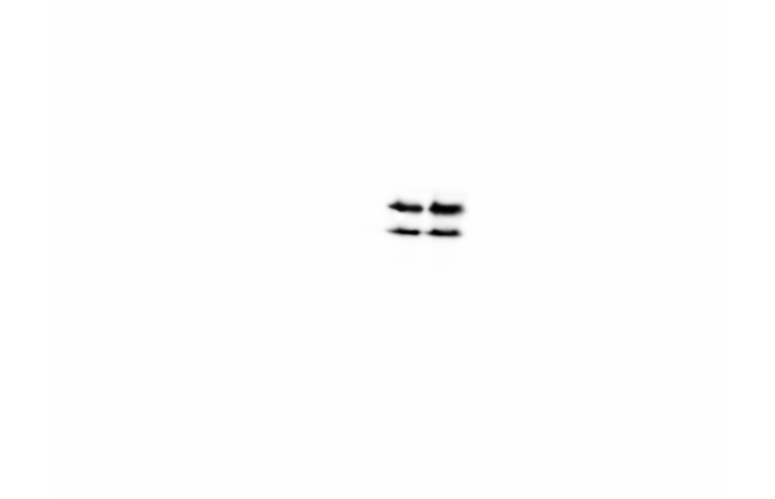

Supplement: S1 File — (ZIP) [file pone.0322326.s006.zip › ú¿6ú⌐WB/╩╡╤Θ╦─ú¿6ú⌐RUNX2 ╫╧═Γ.tif]
