# Supplementary material for: Integrated bulk RNA and single-cell analysis with experimental validation reveal oxidative stress-related diagnostic biomarkers for osteoporosis
Source: PLoS One. 2025 Apr 29;20(4):e0322326. doi: 10.1371/journal.pone.0322326 (PMC12040157; doi:10.1371/journal.pone.0322326)
Supplement: Attachment — Submitted filename: Response to Reviewers.docx [file pone.0322326.s008.docx]

Dear Editors and Reviewer:

Thank you for your letter and for the reviewers’ comments concerning our manuscript entitled “**Integrated Bulk RNA and Single-Cell Analysis with Experimental Validation Reveal Oxidative Stress-Related Diagnostic Biomarkers for Osteoporosis**” (Manuscript Number: PONE-D-24-52627). Those comments are all valuable and very helpful for revising and improving our paper, as well as the important guiding significance to our research. We have studied the comments carefully and have made corrections which we hope meets with approval. Revised portions are shown as TRACK CHANGES in the manuscript. The main corrections in the paper and the point-by-point responses to the reviewer’s comments are as flowing:

***Response to Reviewer#1:***

**Comment 1**: The CHRM2/COL4A2 pathway was found in the paper works very well for the early OP diagnosis. On the other hand, A variety of early indicators for osteoporosis are currently available; where does CHRM2 have its advantages? Please detail this in the results or discussion section.

**Response**: Thank you for this insightful question. While multiple early indicators for OP already exist—such as serum bone turnover markers (e.g., osteocalcin, CTX, and PINP), Wnt/β-catenin-related molecules (e.g., sclerostin), and various microRNAs—our data highlight several unique advantages of **CHRM2**:

1. **Direct Link to Osteogenic Differentiation**
   Unlike many markers that only reflect resorption or turnover, CHRM2 tightly aligns with the osteogenic process, as seen in its temporal expression changes during early to late osteoblast differentiation.
2. **Integration With Immune and Oxidative Stress Pathways**
   CHRM2’s regulatory role in both osteoblast maturation and immune cell interactions (via the CHRM2/COL4A2 pathway) may offer added diagnostic clarity, especially in early disease stages.
3. **Potential Early Detection**
   CHRM2’s dynamic expression pattern may detect subtle bone remodeling shifts before clinical OP manifestations, thus complementing more traditional markers.

We have now added a concise comparison in the **Discussion** (page 19, lines 789–802) to underscore why CHRM2 may serve as a promising early indicator alongside other established pathways. Thank you for your valuable feedback.

**Comment** **2***.* For the above reason, the presentation should be focused on the results. The transitions between each section are rather abrupt, and there are certain logical issues. The connection between the bioinformatics analysis and the subsequent experimental parts is poor; please make revisions.

**Response:** Thank you for your comment regarding the abruptness of the section transitions and the poor connection between the bioinformatics analysis and the subsequent experimental parts. We have reorganized the Results to emphasize how the bioinformatics discoveries—particularly the identification of CHRM2/COL4A2—led us to conduct the follow-up experiments. Specifically, we have integrated clearer explanations of why the bioinformatic data pointed to CHRM2 as a candidate and how this insight shaped the in vitro validation. Additionally, we have used sequencing results to highlight our focus on the regulation of COL4A2 by CHRM2. By presenting the computational findings and experimental rationale in a more straightforward sequence, we believe the manuscript now flows more logically and underlines the significance of the CHRM2/COL4A2 pathway in early osteoporosis detection. We appreciate your feedback and hope these revisions address your concerns.

.

Comment 3. In the cell experimentation section, the authors used siRNA to knock down COL4A2 in stem cells and observed their subsequent osteogenic differentiation ability. They also established a CHRM2 stem cell knockout model. Why not use this model to conduct experiments related to osteogenic differentiation?

Response: Thank you for raising this point. Initially, we focused on siRNA-mediated COL4A2 knockdown to delineate its specific role in osteogenic differentiation. After these experiments demonstrated the negative regulatory effect of COL4A2 on osteogenesis, we established the CHRM2 knockout model to explore the broader implications of the CHRM2/COL4A2 pathway in cell proliferation and osteogenic processes. In the manuscript, we performed Western blot, flow cytometry, ALP activity assay, and Alizarin Red staining experiments to compare the knockdown group with the control group. The experimental results confirmed that CHRM2 is a negative regulator in the osteogenic differentiation process. We appreciate this suggestion and will integrate these experiments into our future work**.**

**Figure 7. The effects of CHRM2 knockdown on osteogenic differentiation and cell proliferation. (A) The expression of CHRM2 was significantly reduced in cells knocked down with Si-CHRM2. (B) The RT-qPCR results showed that the expression of CHRM2 in cells with Si-CHRM2 knockdown decreased significantly. (C) The cell proliferation were measured by OD value at 450 nm, and the observation time points were at 0, 24, 48, 72 and 96 hours. (D) The protein expression levels of osteogenic markers RUNX2, OPN, and Osterix in Si-CHRM2 knockdown cells and control cells by western blot. (E) Flow cytometry of cell cycle in the control group and the experimental group. (F) ALP staining in the Si-CHRM2 experimental group. (G) Alizarin red staining. *p < 0.05; **p < 0.01; ***p < 0.001; ns: no significance.**

Comment 4. A few sentences on the organization of the paper will be helpful in line 193-233.

Response: Thank you for pointing this out. This new text provides a concise overview of the paper’s organization, showing how each section builds on the previous one. We believe this adjustment will help readers follow the logical progression from data analysis to experimental validation more smoothly.

Comment 5. The clarity of the figures in the article is poor; please replace them with clearer ones.

Response: Thank you for bringing the figure clarity to our attention. We have replaced the original figures with higher-resolution versions (at least 300 dpi) and adjusted the formatting to improve visibility and detail. We hope these revised figures will better convey our results and enhance the overall readability of our manuscript.

Comment 6. The English of your manuscript must be improved before resubmission. We strongly suggest that you obtain assistance from a colleague who is well-versed in English or whose native language is English.

Response: Thank you for your recommendation. We have thoroughly revised the manuscript with the assistance of a native English speaker to ensure accuracy, clarity, and fluency. We appreciate your feedback and believe that our revisions address the language concerns. If there are any additional improvements you would suggest, we would be glad to make further adjustments.

***Response to Reviewer#2:***

**Comment 1:** In the Discussion section, although the research results were elaborated in relatively detailed manner, the explanations for some experimental results (such as the reasons for the expression changes of certain genes at different time points) were not in-depth and comprehensive enough. It is recommended to further combine relevant literature and research progress to conduct a more in-depth analysis and discussion on the experimental results.

Response: Thank you for your valuable comments regarding the depth and comprehensiveness of the Discussion section, especially concerning the temporal changes in gene expression. We have revised the Discussion to include additional literature and a more thorough analysis of the molecular mechanisms behind these observations.

We appreciate the reviewer’s feedback and trust that these revisions and additional references will address the request for a more comprehensive and literature-supported discussion of our time-course findings.

Comment 2: Some sentences were not expressed clearly and smoothly enough, and there were some grammatical errors and improper use of professional terms. It is recommended to carefully proofread and modify them to improve the readability of the article.

Response: Thank you for highlighting the need for clearer language and more precise terminology. We have meticulously reviewed and revised the manuscript to address grammatical issues and refine the use of specialized terms, ensuring that our phrasing is both accurate and easily understood by readers. We appreciate your feedback and believe these improvements enhance the overall clarity and readability of our work.

Comment 3: The labels and descriptions of some charts and figures were not detailed enough. For example, some gene names and abbreviations were not explained in the charts and figures, which affected readers' understanding of the information in the charts and figures. It is recommended to optimize and improve the charts and figures.

Response: Thank you for pointing out the need for more detailed labeling and annotation in our charts and figures. We have revised the figure legends to include clear explanations of all gene names, abbreviations, and relevant symbols. Additionally, we have updated the figure labels to provide sufficient detail for readers to interpret the data accurately. We appreciate your feedback and believe these adjustments will improve the clarity and accessibility of our visual materials.
